# Prediction of Mild Cognitive Impairment Conversion Using a Combination of Independent Component Analysis and the Cox Model

**DOI:** 10.3389/fnhum.2017.00033

**Published:** 2017-02-06

**Authors:** Ke Liu, Kewei Chen, Li Yao, Xiaojuan Guo

**Affiliations:** ^1^College of Information Science and Technology, Beijing Normal UniversityBeijing, China; ^2^Banner Alzheimer’s Institute and Banner Good Samaritan PET Center, PhoenixAZ, USA

**Keywords:** Cox model, independent component analysis, mild cognitive impairment, structural MRI, FDG-PET

## Abstract

Mild cognitive impairment (MCI) represents a transitional stage from normal aging to Alzheimer’s disease (AD) and corresponds to a higher risk of developing AD. Thus, it is necessary to explore and predict the onset of AD in MCI stage. In this study, we propose a combination of independent component analysis (ICA) and the multivariate Cox proportional hazards regression model to investigate promising risk factors associated with MCI conversion among 126 MCI converters and 108 MCI non-converters from the Alzheimer’s Disease Neuroimaging Initiative (ADNI) database. Using structural magnetic resonance imaging (MRI) and fluorodeoxyglucose positron emission tomography (FDG-PET) data, we extracted brain networks from AD and normal control groups via ICA and then constructed Cox models that included network-based neuroimaging factors for the MCI group. We carried out five separate Cox analyses and the two-modality neuroimaging Cox model identified three significant network-based risk factors with higher prediction performance (accuracy = 73.50%) than those in either single-modality model (accuracy = 68.80%). Additionally, the results of the comprehensive Cox model, including significant neuroimaging factors and clinical variables, demonstrated that MCI individuals with reduced gray matter volume in a temporal lobe-related network of structural MRI [hazard ratio (HR) = 8.29E-05 (95% confidence interval (CI), 5.10E- 07 ~ 0.013)], low glucose metabolism in the posterior default mode network based on FDG-PET [HR = 0.066 (95% CI, 4.63E-03 ~ 0.928)], positive apolipoprotein E ε4-status [HR = 1. 988 (95% CI, 1.531 ~ 2.581)], increased Alzheimer’s Disease Assessment Scale-Cognitive Subscale scores [HR = 1.100 (95% CI, 1.059 ~ 1.144)] and Sum of Boxes of Clinical Dementia Rating scores [HR = 1.622 (95% CI, 1.364 ~ 1.930)] were more likely to convert to AD within 36 months after baselines. These significant risk factors in such comprehensive Cox model had the best prediction ability (accuracy = 84.62%, sensitivity = 86.51%, specificity = 82.41%) compared to either neuroimaging factors or clinical variables alone. These results suggested that a combination of ICA and Cox model analyses could be used successfully in survival analysis and provide a network-based perspective of MCI progression or AD-related studies.

## Introduction

Alzheimer’s disease (AD) is one of the most severe neurodegenerative diseases and is accompanied by structural and functional changes in the brain (Brookmeyer et al., 2007; Dartigues, 2009; Jack et al., 2010a; Reitz et al., 2011; Prince, 2015). Mild cognitive impairment (MCI), which is a transitional stage between normal aging and AD, is associated with a higher risk of developing AD (Albert et al., 2011; Roberts and Knopman, 2013). Thus, it is necessary to explore and predict the onset of AD at the MCI stage.

Many studies have documented that MCI patients exhibit cognitive impairments and neurological changes (Karas et al., 2004; Farias et al., 2006; Dickerson and Sperling, 2008; Schneider et al., 2009; Morbelli et al., 2010; Wee et al., 2012). For example, compared to the Clinical Dementia Rating (CDR) scores of cognitively normal controls (NC), the CDR scores of MCI individuals increased from 0 to 0.5 or even 1.0 (Cedarbaum et al., 2013; Williams et al., 2013). In addition, brain gray matter volume reduction, hypometabolism, and amyloid-beta (Aβ) deposition are apparent in MCI individuals and have been effectively detected by neuroimaging techniques (Jack et al., 1999, 2010b; Devanand et al., 2010; Sluimer et al., 2010). Thus, the use of cognitive test scores and neuroimaging biomarkers to predict MCI conversion has been brought to the forefront (Bischkopf et al., 2002; Petersen, 2002; Grundman et al., 2004; Misra et al., 2009; Jessen et al., 2014).

Survival analysis is a statistical method used to analyze survival data in consideration of censored data and survival time on event occurrence (Cox and Oakes, 1984; Cox, 1992). The multivariate Cox proportional hazards regression model is one of the most popular semiparametric models in survival analysis. It is used to estimate the relationship between risk factors and survival time or other censored outcomes, as part of efforts to understand the risk factors that may have potential roles in preventing or delaying the onset of disease (D’Amico et al., 2000; Partridge et al., 2005; Li et al., 2013). Recently, multivariate Cox models, including cognitive test scores or neuroimaging biomarkers as covariates, have been increasingly used for the early identification and prognosis of patients who progress from MCI to AD (Desikan et al., 2010; Chen et al., 2011; Li et al., 2013; Egli et al., 2014; Moradi et al., 2015; Zeifman et al., 2015). Egli et al. (2014) investigated which cognitive variables were best predictors for progression to AD from MCI within 36-month observation period, and suggested that serial position scores predicted MCI conversion with survival time longer than 18 months. By building Cox models based on gray matter density, Moradi et al. (2015) found the voxels that had a higher accuracy for predicting MCI conversion were mainly located in the hippocampus, the temporal and frontal lobes, and the cerebellar areas. Chen et al. (2011) characterized AD patients with a hypometabolic convergence index (HCI) from fluorodeoxyglucose positron emission tomography (FDG-PET) data, and the results of the Cox model demonstrated that MCI patients with a higher HCI had a hazard ratio (HR) of 6.55 for conversion to probable AD within 18 months. Multimodal neuroimaging studies took advantage of the complementary information provided by different brain imaging modalities used in AD identification or classification, as biomarkers from different modalities reflect different aspects of brain changes (Li et al., 2008; Yuan et al., 2009; Jack et al., 2010b; Wee et al., 2012; Dickerson et al., 2013). Jack et al. (2010b) combined the hippocampal volume of structural magnetic resonance imaging (MRI) and Aβ load biomarker in a Cox model to evaluate the ability of these two factors in predicting MCI progression over 3-year follow-up and found that MCI individuals with higher Aβ load level and smaller hippocampal volumes are more likely to convert to AD.

Previous brain imaging studies constructed multivariate Cox models to explore the best predictors based on the extracted neuroimaging features from brain regions of interest (ROIs) (Jack et al., 1999; Li et al., 2008; Yuan et al., 2009; Wee et al., 2012) or the whole brain (voxel-level analysis) (Chen et al., 2011; Vemuri et al., 2011; Zeifman et al., 2015). However, ROI analysis depends mostly on a priori knowledge. Voxel-level analysis takes full advantage of information across the whole brain, but modeling based on each voxel usually brings in onerous computing workload. Independent component analysis (ICA) is a powerful multivariate method for use in blind source separation problems to extract maximally independent components (ICs) or sources from a mixed signal (Hyvärinen and Oja, 2000). It has been suggested that neurological changes in different voxels or regions of the human brain exhibited covariance, and it is also impractical to include voxel-level information in Cox model analysis due to the huge number of predictors. ICA is one approach that integrates voxel-wise information into a few ICs, but also utilizes the inter-regional covariance relationships among the whole brain. By considering imaging data to be linear combinations of statistically independent sources, ICA has been widely used to investigate brain structural or functional networks in different populations (Beckmann et al., 2005; Mantini et al., 2007; Segall et al., 2012; Hafkemeijer et al., 2014). The voxels within such networks carry similar covariate information (Xu et al., 2009). Therefore, a combination of ICA and the multivariate Cox proportional hazards regression model could provide a network-based perspective to analyze survival data of MCI individuals and predict MCI conversion.

The present study aimed to investigate promising risk factors and to analyze their effects on MCI conversion by combining ICA and the multivariate Cox proportional hazards regression model. We first applied ICA to extract brain networks from structural MRI and FDG-PET images in AD and NC groups, respectively. Then, the mask images of the brain networks that exhibited significant between-group differences were generated to extract and compute independent variates of MCI baseline neuroimaging data. Finally, multivariate Cox proportional hazards regression models consisting of different types of covariates among MCI individuals were constructed.

## Materials and Methods

### Alzheimer’s Disease Neuroimaging Initiative

The data used in this study were obtained from the Alzheimer’s Disease Neuroimaging Initiative (ADNI) database^1^. The ADNI was launched in 2003 as a public–private partnership, led by Principal Investigator Michael W. Weiner, MD. The primary goal of the ADNI has been to test whether serial MRI, PET, other biological markers, and clinical and neuropsychological assessments could be combined to measure the progression of MCI and early AD. For up-to-date information, see www.adni-info.org.

### Participants

This study included two independent cohorts: group 1 (121 AD patients and 120 NC subjects) and group 2 [126 MCI converters (MCI-c) and 108 MCI non-converters (MCI-nc)] from the ADNI database. All 475 subjects had both structural MRI and FDG-PET data.

Group inclusion criteria were as follows. NC subjects had no memory complaints, a CDR score of 0 and Mini-Mental State Examination (MMSE) scores between 26 and 30. AD patients had memory complaints, CDR scores between 0.5 and 2.0, and MMSE scores less than 26, and they met the criteria for probable AD diagnosis according to the National Institute of Neurological and Communicative Disorders and Stroke/Alzheimer’s Disease and Related Disorders Association (NINCDS/ADRDA; McKhann et al., 1984). MCI subjects had a CDR score of 0.5, MMSE scores between 21 and 30, as well as memory complaints and abnormal memory function according to the Logical Memory II subscale (Delayed Paragraph Recall), but an absence of dementia. The MCI patients who progressed to AD within 3 years upon follow-up were classified into the MCI-c group; patients who maintained a diagnosis of MCI or those who were censored during the same time interval were classified into the MCI-nc group. The AD group did not significantly differ from the NC group in gender ratio (*p* = 0.14) or age (*p* = 0.68), but exhibited significantly lower MMSE scores (*p* = 1.25E-42). In addition, there were no significant differences between the MCI-c and MCI-nc groups with respect to gender ratio (*p* = 0.66) or age (*p* = 0.20). **Table 1** shows the detailed clinical and demographic information for AD, NC, and MCI subjects. **Supplementary Table S1** provides a list of subjects’ ID.

**Table 1:** The clinical and demographic characteristics of participants with AD, NC, MCI-c, and MCI-nc groups.

	AD (*n* = 121)	NC (*n* = 120)	MCI-c (*n* = 126)	MCI-nc (*n* = 108)
Age (years)	74.87 ± 8.07	75.26 ± 6.52	73.47 ± 7.23	73.33 ± 7.73
Gender (M/F)	70/51	58/62	77/49	69/39
Education (years)	15.72 ± 2.61	16.43 ± 2.74	16.09 ± 2.64	15.89 ± 2.63
MMSE score	21.71 ± 3.94	29.18 ± 0.98	26.88 ± 1.76	28.06 ± 1.75
APOE ε4 (NC/HT/HM)	41/80/0	79/33/8	37/65/24	67/35/6
ADAS-cog score	21.52 ± 7.96	5.76 ± 3.02	13.60 ± 4.64	8.03 ± 3.47
Conversion time (years)	–	–	1.48 ± 0.69	–


*AD, Alzheimer’s disease; NC, normal control; MCI, mild cognitive impairment; MCI-c, MCI converter; MCI-nc, MCI non-converter; M/F, male/female; MMSE, Mini-Mental State Examination; APOE, apolipoprotein E; NC, non-carrier; HT, heterozygote; HM, homozygote; ADAS-cog, Alzheimer’s Disease Assessment Scale-Cognitive Subscale.*

### Neuroimaging Data Acquisition

#### Structural MRI Data

Structural MRI images were acquired at multiple sites with different acquisition parameters. The scanning parameters can be found at http://adni.loni.usc.edu/methods/documents/mri-protocols/. For each participant, a T1-weighted magnetization-prepared rapid gradient echo (MPRAGE) image was acquired on 1.5 T or 3 T scanners. The structural MRI scans had undergone certain intensity non-uniformity and gradient non-linearity correction, such as gradwarp, B1 calibration and N3 correction. Details can be found at http://adni.loni.usc.edu/methods/mri-analysis/mri-pre-processing/. For each subject, the processed NIFTI images were downloaded.

#### FDG-PET Data

Subjects were asked to abstain from all food and fluids (except water) from midnight the night before the scan until either after the imaging was completed or for at least 2 h prior to the FDG-PET imaging session. After mandatory confirmation of compliance to the dietary requirements, the baseline blood glucose level was measured. Then, subjects were injected with 185 MBq (5 ± 0.5 mCi) of [18F]-FDG. Subsequently, subjects were allowed to rest comfortably for approximately 20 min for the incorporation of [18F]-FDG. Finally, a dynamic 3D scan with six 5-min frames was acquired.

For FDG-PET data, all separate temporal frames were co-registered to the first frame of the raw image file to lessen the effects caused by patients’ head motion. Then, these co-registered frames were averaged to create a single image. After being co-registered and averaged, each FDG-PET image was reoriented into a standard image grid and the size of voxels became 1.5 mm cubic. Finally, an 8 mm full-width-at-half-maximum (FWHM) Gaussian kernel was used to smooth the above-mentioned images. Details about these four steps of processed PET image data can be found at http://adni.loni.usc.edu/methods/pet-analysis/pre-processing/. In this study, images labeled with “Coreg, Avg, Std Img and Vox Siz, Uniform Resolution” were downloaded.

### Image Preprocessing

The spatial preprocessing of all brain images was implemented in Statistical Parametric Mapping (SPM8)^2^.

The segmentation and normalization of structural MRI images were performed using the Voxel-Based Morphometry (VBM) Toolbox^3^. First, each structural MRI image was segmented based on an adaptive maximum a posteriori (MAP) and a partial volume estimation (PVE) approach (Rajapakse et al., 1997; Tohka et al., 2004). Two de-noising methods were implemented during segmentation, a spatially adaptive non-local means (SANLM) de-noising filter and a classical Markov Random Field (MRF) approach (Rajapakse et al., 1997; Manjón et al., 2010). Then, gray matter images were normalized using a high-dimensional protocol called Diffeomorphic Anatomical Registration using Exponential Lie Algebra (DARTEL; Ashburner, 2007). During normalization, the creation of the template and the registration of the image were performed iteratively. Finally, gray matter images were transformed to the Montreal Neurological Institute (MNI) space and were spatially smoothed with a 3D Gaussian kernel with 8-mm FWHM.

FDG-PET images were first coregistered to each individual’s structural MRI image and were then normalized to the MNI space with the corresponding normalization parameters derived from the above DARTEL procedures. By normalizing to the mean uptake of the global cerebrum, standard uptake value ratio (SUVr) images were calculated (Jagust et al., 2010). These SUVr images and smoothed gray matter images were used for subsequent analysis.

### ICA Analysis

The ICA was implemented using the Fusion ICA toolbox (FIT)^4^. For the AD and NC groups, gray matter images and SUVr images were analyzed separately. Using the Infomax algorithm, the initial structural MRI data matrix (subjects by voxels) of AD and NC groups was decomposed into a mixing coefficient matrix (subjects by sources) and a source matrix (sources by voxels). The optimal numbers of ICs (the source matrix’s row) were estimated based on the Minimum Description Length (MDL) criteria. A two sample *t*-test was performed on each column of the mixing coefficient matrix (ICA weights of each IC), which represented the degree to which one subject contributed to the corresponding source network, to evaluate the difference between AD and NC groups for each IC. Only ICs whose ICA weights showed significant between-group differences after Bonferroni correction were converted to *Z*-score maps and then transferred into 3D brain maps. Subsequently, binarization templates of structural MRI brain networks were generated with the threshold of *Z* ≥ 3.0. For MCI subjects, we calculated the average gray matter volume based on the voxels within each binarization network template in the individual’s structural MRI image as the structural MRI neuroimaging factors in the Cox model. The neuroimaging factors of FDG-PET SUVr images were generated using the same method as the structural MRI images.

### Cox Model Analysis

For each MCI individual, time “0” was defined as the date of the baseline assessment. The initial event was considered the diagnosis of MCI, and the endpoint event was considered the conversion to AD. Survival time was evaluated by the month. For MCI-c subjects, it was defined as the time from the baseline scan to the diagnosis of AD. For MCI-nc subjects who were censored at the last follow-up, survival time was 36 months in this study. The covariates in the Cox models mainly consisted of brain neuroimaging factors extracted from structural MRI and FDG-PET data and clinical variables, such as the genetic status [apolipoprotein E (APOE) ε4-status], age at baseline scan (years), gender, education (years), MMSE scores, Alzheimer’s Disease Assessment Scale-Cognitive Subscale (ADAS-cog) scores and Sum of Boxes of CDR (CDR-SB) scores. The HR from the Cox model analysis indicated the change in the risk of progressing to AD caused by the per 1 unit change in the corresponding covariate. An HR smaller than 1 or a β value (the regression coefficient) less than 0 indicated that a smaller value of the covariate was associated with a shorter time or greater risk to MCI progression.

We carried out five separate Cox analyses. Initially, two single-modality Cox models were constructed for structural MRI and FDG-PET imaging data. Then, the neuroimaging factors that significantly predicted the conversion of MCI in the single-modality analysis were entered into the two-modality Cox model analysis. In addition, we built a Cox model consisting of clinical variables. Finally, we entered both of these significantly predictive neuroimaging factors and clinical variables into a comprehensive Cox model to evaluate the effects of the comprehensive predictors on MCI conversion to AD.

Finally, the area under the curve (AUC), sensitivity, specificity and accuracy were calculated via the receiver operating characteristic (ROC) curve analysis to assess the prediction abilities of these significant factors in the Cox models. In the ROC curve analysis, the logistic regression analysis was used to combine those significant risk factors from the Cox model and generate a predicted value as a new index. The AUC closer to 1 indicated that the index had higher diagnostic value.

## Results

The number of estimated ICs was 49 for structural MRI data and 33 for FDG-PET data in the AD and NC groups, respectively. After Bonferroni correction, 21 and 13 ICs showed significant between-group differences.

Results of the single-modality Cox model showed that the average gray matter volume and the SUVr of several brain networks, such as IC_06 (HR = 2.40E-07) and IC_47 (HR = 2.56E-06) of structural MRI, and IC_27 (HR = 5.26E-04), IC_28 (HR = 1.39E-03) of FDG-PET (**Tables 2** and **3**), had significant effects on progression from MCI to AD. The prediction accuracy of risk factors from the structural MRI was accuracy = 68.80%, AUC = 0.748, sensitivity = 64.29%, specificity = 74.07%, and accuracy = 68.80%, AUC = 0.736, sensitivity = 57.14%, and specificity = 82.41% for FDG-PET (**Table 4**).

**Table 2:** The results of the Cox model analysis.

Covariates of the Cox model		β	SE	*p*-value	HR (95% CI)
**Single-modal neuroimaging factors**					
Structural MRI	IC_06	-15.245	5.470	5.32E-03	2.40E-07 (5.29E-12, 0.011)
	IC_47	-12.874	4.194	2.14E-03	2.56E-06 (6.90E-10, 0.095)
FDG-PET	IC_27	-7.550	1.535	8.72E-07	5.26E-04 (2.60E-05, 0.011)
	IC_28	-6.580	1.766	1.95E-04	1.39E-03 (4.35E-05, 0.044)
**Two-modality neuroimaging factors**					
Structural MRI	IC_06	-8.459	2.093	5.33E-05	2.12E-04 (3.50E-06, 0.013)
FDG-PET	IC_27	-6.600	1.266	1.88E-07	1.36E-03 (1.14E-04, 0.016)
	IC_28	-5.000	1.528	1.07E-03	6.74E-03 (3.37E-04, 0.135)
**Clinical variables**					
	ADAS-cog	0.130	0.019	2.14E-11	1.139 (1.097, 1.184)
	CDR-SB	0.431	0.082	1.64E-07	1.538 (1.309, 1.808)
	APOE ε4	0.633	0.135	2.81E-06	1.882 (1.445, 2.453)
**Neuroimaging factors and clinical variables**					
	ADAS-cog	0.096	0.020	1.23E-06	1.100 (1.059, 1.144)
	CDR-SB	0.484	0.089	4.82E-08	1.622 (1.364, 1.930)
	APOE ε4	0.687	0.133	2.48E-07	1.988 (1.531, 2.581)
Structural MRI	IC_06	-9.398	2.598	2.98E-04	8.29E-05 (5.10E-07, 0.013)
FDG-PET	IC_27	-2.724	1.352	0.044	0.066 (4.63E-03, 0.928)


*IC, independent component; β, the regression coefficient; SE, standard error; HR, hazard ratio; CI, confidence interval.*

**Table 3:** Brain regions within brain networks with significant prediction value in single-modality Cox models.

Brain regions	Peak coordinates			*Z*	Cluster size
	MNI (*X. Y. Z*)				(mm^3^)
**Structural MRI: IC_06**					
L middle temporal gyrus	-57	-56	-5	18.81	17071
R middle temporal gyrus	60	-41	-14	9.87	2865
L inferior temporal gyrus	-57	-56	-6	18.31	10641
R inferior temporal gyrus	60	-44	-12	10.39	3318
L middle occipital gyrus	-53	-68	-2	9.94	9362
**Structural MRI: IC_47**					
L hippocampus	-23	-9	-21	16.19	5943
R hippocampus	24	-6	-23	20.15	5943
L parahippocampal gyrus	-21	-8	-26	14.18	6267
R parahippocampal gyrus	24	-6	-24	19.48	6689
**FDG-PET: IC_27**					
L precuneus	-3	-66	32	10.44	12508
R precuneus	2	-65	35	10.31	12855
L middle cingulate gyrus	0	-48	35	8.73	3810
R middle cingulate gyrus	2	-54	32	10.11	3976
L posterior cingulate gyrus	0	-54	30	10.27	2936
R posterior cingulate gyrus	2	-54	30	10.25	1593
**FDG-PET: IC_28**					
L inferior temporal gyrus	-45	-3	-42	6.76	9966
R inferior temporal gyrus	45	-15	-36	7.15	11421
L fusiform gyrus	-30	-12	-36	7.05	4833
R fusiform gyrus	44	-17	-36	6.88	5241


*L, left; R, right; coordinates in MNI space.*

**Table 4:** The prediction accuracy of the significant covariates.

Covariates of the Cox model	Accuracy (%)	Sensitivity (%)	Specificity (%)	AUC
**Neuroimaging factors**				
Structural MRI	68.80	64.29	74.07	0.748
FDG-PET	68.80	57.14	82.41	0.736
Structural MRI and FDG-PET	73.50	76.19	70.37	0.808
**Clinical variables**	81.62	77.78	86.11	0.888
**Neuroimaging factors and clinical variables**	84.62	86.51	82.41	0.920


*AUC, area under the curve.*

The results of the two-modality Cox model showed that IC_06 (HR = 2.12E-04) for structural MRI, IC_27 (HR = 1.36E-03) and IC_28 (HR = 6.47E-03) for FDG-PET were associated with MCI conversion (**Table 2**). The prediction accuracy was accuracy = 73.50%, AUC = 0.808, sensitivity = 76.19%, and specificity = 70.37% (**Table 4**).

Regarding the Cox model that included all clinical variables as covariates, ADAS-cog scores (HR = 1.139), CDR-SB scores (HR = 1.538) and APOE ε4-status (HR = 1.882) were significant for the progression from MCI to AD (**Table 2**). The prediction accuracy was accuracy = 81.62%, AUC = 0.888, sensitivity = 77.78%, and specificity = 86.11% (**Table 4**).

**FIGURE 1:**
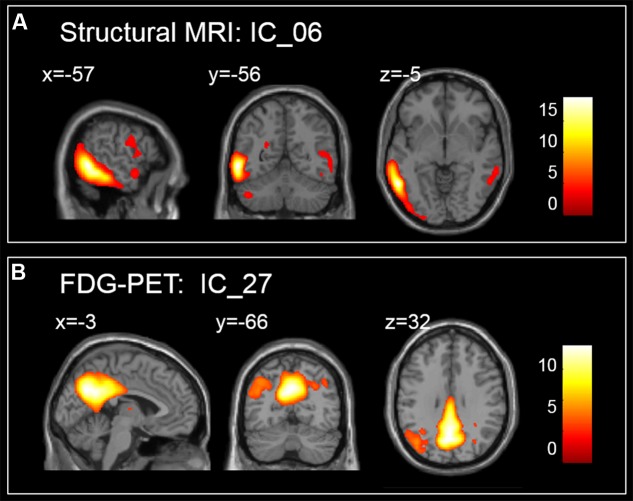
**Maps of brain networks with significant risk in a comprehensive Cox model,** **(A)** for IC_06 of structural MRI and **(B)** for IC_27 of FDG-PET.

**FIGURE 2:**
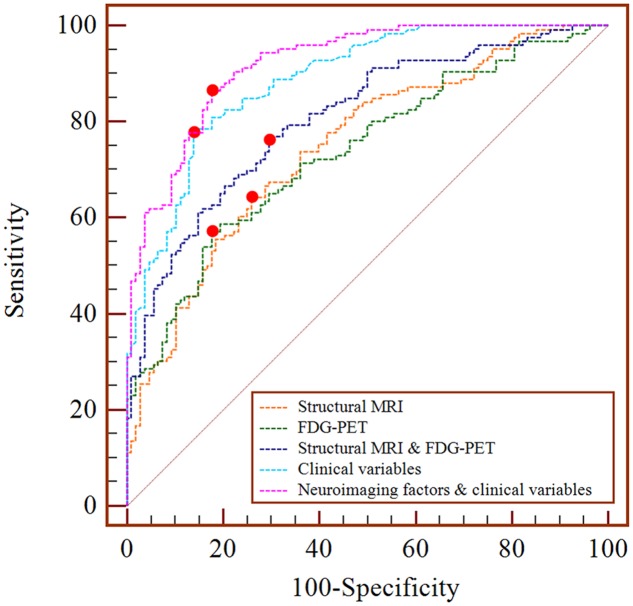
**ROC curves of the significant covariates in each Cox model**.

When both the significant risk factors of the two-modality model and the clinical variables were entered into a comprehensive Cox model, IC_06 for structural MRI [HR = 8.29E-05 (95% confidence interval (CI), 5.10E-07 ∼ 0.013)] and IC_27 for FDG-PET [HR = 0.066 (95% CI, 4.63E-03 ∼ 0.928)], ADAS-cog scores [HR = 1.100 (95% CI, 1.059 ∼ 1.144)], CDR-SB scores [HR = 1.622 (95% CI, 1.364 ∼ 1.930)] and positive APOE ε4-status [HR = 1. 988 (95% CI, 1.531 ∼ 2.581)] were the most predictive of MCI conversion (**Table 2**). **Figure 1** shows the maps of these two brain networks (IC_06 of structural MRI and IC_27 of FDG-PET). The prediction accuracy was accuracy = 84.62%, AUC = 0.920, sensitivity = 86.51%, and specificity = 82.41% (**Table 4**). **Figure 2** shows the ROC curves of the significant variates in each Cox model. IC_06 for structural MRI predominantly contained the bilateral middle and inferior temporal gyrus and the left middle occipital gyrus. Structural MRI IC_47 primarily included the bilateral hippocampus and parahippocampal gyrus. For FDG-PET, IC_27 consisted of the bilateral precuneus and the middle and posterior cingulate gyri. IC_28 encompassed the bilateral inferior temporal gyrus and fusiform gyrus. The main brain clusters in each network are described in **Table 3**.

## Discussion

In this study, we first identified the ICs from AD and NC groups via ICA and extracted the neuroimaging factors from individuals with MCI. Then, multivariate Cox proportional hazard regression models were performed to evaluate the influence of predictors of interest on the time to onset of AD dementia among MCI individuals. We found that reduced gray matter volume in structural MRI images, low glucose metabolism according to FDG-PET, increased ADAS-cog scores and CDR-SB scores, and a positive APOE ε4-status had significant effects on the progression of MCI to AD.

### Significant Effect of Single-Modality Analysis

Based on the single-modality data, the mean gray matter volumes of IC_06 (HR = 2.40E-07, *p* = 5.32E-03) and IC_47 (HR = 2.56E-06, *p* = 2.14E-03) based on structural MRI were significant risk factors for MCI conversion. An HR smaller than 1 means that reduced gray matter volume in these two ICs gave rise to a higher risk of progression to AD. Primarily contained within the bilateral middle and inferior temporal gyrus, IC_06 was recognized as a temporal lobe-related network. Structural MRI IC_47 was considered a hippocampus-related network linked to memory (Wagner et al., 1998; Celone et al., 2006). The results of the FDG-PET Cox model analysis showed that the glucose metabolism in IC_27 (HR = 5.26E-04, *p* = 8.72E-07) and IC_28 (HR = 1.39E-03, *p* = 1.95E-04) were significantly related to MCI-to-AD conversion. IC_27 and IC_28 were considered to be the posterior default mode network (DMN) and a temporal lobe-related network, respectively. The HR value suggested that lower glucose metabolism significantly affected MCI conversion. As shown in **Table 2**, the HRs of ICs differed from each other, which suggested that the neurological changes in different brain networks represented different degrees of risk for MCI progression. The main brain structural regions within significant predictive networks identified in the current study are consistent with reports in the literature (Jack et al., 2000; Desikan et al., 2010; Devanand et al., 2012; Prestia et al., 2013; Zeifman et al., 2015). For example, Jack et al. (2000) found that the annual rates of hippocampal volume loss in MCI decliners group was significantly greater than in stable MCI group which indicated that hippocampal atrophy was correlated with AD-related changes in clinical status. Zeifman et al. (2015) analyzed MRI scans of 58 incident MCI patients, 151 AD patients, and 292 cognitively normal participants and fitted per-voxel Cox proportional hazard models to examine the effects of gray matter volume on the time to develop MCI or AD from normal cognition. They found that voxels located within three brain regions were significantly associated with time to AD: the mesial temporal lobe, including the anterior hippocampus extending into the amygdala, and the posterior cingulate gyrus. In addition, voxels in the anterior hippocampus/amygdala were also associated with progression from NC to MCI (Zeifman et al., 2015).

Several studies have documented that gray matter atrophy or hypometabolism were associated with an increased risk of progression to AD (Yetkin et al., 2006; Tapiola et al., 2008; Devanand et al., 2012; Koch et al., 2012; Prestia et al., 2013; Zeifman et al., 2015). Based on the brain structural MRI data, Devanand et al. (2012) considered the signed euclidean distances at each voxel into linear regression models for baseline analysis and constructed Cox models in the MCI sample (31 converters to AD, 99 non-converters). In that study, atrophy of hippocampus for MCI-c was more considerable than MCI-nc and atrophy of the parahippocampal gyrus was also risk factors with relative moderate robustness for MCI conversion (Devanand et al., 2012). Our findings of single modality Cox models suggested that the hippocampus-related network containing both hippocampus and parahippocampal gyrus was also a risk factor and it indicated that a brain network respective could be considered one of the possible attempts to investigate the risk factors about MCI progression. Prestia et al. (2013) adopted the multivariate general linear model and compared the sensitivity and specificity of AD-related biomarkers including the hippocampal volume, cerebrospinal fluid (CSF) biomarkers, and three FDG-PET indices of hypometabolism, the PMOD Alzheimer’s discrimination analysis tool (PALZ), the HCI, the hippocampal volume and the meta-ROI average based on prodromal AD and stable MCI patients from ADNI and Translational Outpatient Memory Clinic database. The diagnostic accuracy of three FDG-PET indices was 52, 61, and 52%, respectively, and 56% for the automatically computed high hippocampal volume, 63% for the semi-automatically computed high hippocampal volume in prodromal AD and stable MCI patients from ADNI (Prestia et al., 2013). Although our study incorporated different models and populations compared with Prestia et al. (2013), our findings also suggested that biomarkers of FDG-PET or structural MRI could be used in predicting MCI conversion. In our single-modality Cox model analysis, FDG-PET showed a similar predictive power as structural MRI, which was in line with the results of the meta-analysis and meta-regression performed by Yuan et al. (2009) which evaluated the ability of FDG-PET, single-photon emission computed tomography (SPECT), and structural MRI imaging to predict MCI conversion and found that FDG-PET performs slightly better.

### Significant Effect of Two-Modality Analysis

When the significant neuroimaging factors in the single-modality analysis were entered into the two-modality Cox model, only structural MRI IC_06, FDG-PET IC_27 and IC_28 were still significant. By employing factor analyses and Cox proportional hazards models based on the baseline MRI scans, Desikan et al. (2010) identified the predictive power of a set of neuroanatomic regions from two ADNI samples, a training cohort (60 MCI-c and 102 MCI-nc) and a validation cohort (58 MCI-c and 104 MCI-nc). The prediction accuracy of MRI-derived factors for the training cohort was AUC = 0.82, sensitivity = 74%, and specificity = 84%; the prediction accuracy for the validation cohort was AUC = 0.84, sensitivity = 87%, and specificity = 66%. When MRI, CSF, and FDG-ROI predictive measures were all included in a Cox model, the prediction accuracy was AUC = 0.83, sensitivity = 90%, and specificity = 69% (Desikan et al., 2010). Our findings from the two-modality Cox model (accuracy = 73.50%, AUC = 0.808, sensitivity = 76.19%, and specificity = 70.37%) were comparable to those of Desikan et al. (2010). Our two-modality Cox model had a higher accuracy than the single-modality model (**Table 4**), which indicated that two-modality neuroimaging factors were more precise than single-modality factors for estimating the risk associated with MCI progression. The two-modality Cox model incorporated complementary information between different brain imaging data. As brain neurological changes of MCI can be detected by neuroimaging techniques effectively, such as the gray matter atrophy by structural MRI and hypometabolism by FDG-PET. It was consistent with other studies, the multimodal Cox model exhibited superior performance compared to the single modality model (Jack et al., 2010b; Chen et al., 2011; Dickerson et al., 2013). Jack et al. (2010b) also performed Cox proportional hazards models to estimate the effects of the Aβ load and hippocampal volume on MCI progression. Their results indicated that both Aβ load [HR = 2.6 (95% CI, 1.5 ∼ 4.5)] and hippocampal volume [HR = 2.6 (95% CI, 1.8∼3.8)] were highly associated with MCI conversion and had the comparable discriminative power when were combined (Jack et al., 2010b).

### Significant Effect of Clinical Variables Analysis

The third Cox model was constructed to include only clinical variables. The results showed that MCI individuals with higher ADAS-cog scores, CDR-SB scores and a positive APOE ε4-status had a higher HR of converting to AD, while age at the time of the baseline scan (years), gender, level of education (years), and MMSE scores were not significant risk factors. A few studies related to MCI conversion have addressed the effects of cognitive variables (Corder et al., 1993; Cedarbaum et al., 2013; Li et al., 2013; Williams et al., 2013; Egli et al., 2014). Egli et al. (2014) constructed Cox models to analyze nine measurements that assessed learning, memory, language, and executive function in 75 MCI individuals. To avoid introducing multicollinearity, correlative variables were entered into separate Cox regression model analyses. The authors then investigated which cognitive variables could predict conversion time longer than 18 months during the 3-year follow-up and found that the serial position scores and Short Delay Free Recall were the best prediction indices (Egli et al., 2014). To explore the utility of CDR-SB as an outcome measure for AD, Cedarbaum et al. (2013) analyzed the internal consistency, structural validity, and other psychometric properties of CDR-SB scores about 382 subjects from ADNI and demonstrated that the CDR-SB scores could be used to assess cognitive and functional conditions in AD patients. We also illustrated that CDR-SB score was a significant risk factor in MCI-to-AD progression from another aspect. Moreover, many studies have suggested that the APOE ε4 gene is a potent genetic risk factor for sporadic and late onset familial AD (Trachtenberg et al., 2012; Murphy et al., 2013; Risacher et al., 2015). Murphy et al. (2013) fitted a linear mixed effects model to analyze brain imaging data and the APOE ε4 status of 194 NC subjects, 212 early MCI subjects, 132 late MCI subjects, and 64 AD subjects and their results showed a significantly observable effect of APOE ε4 (Cohen’s *d* = 0.96) on Aβ plaque density which rose dramatically in AD comparing to NC (Murphy et al., 2013). In our study, MCI individual who had a positive APOE ε4 status at baseline suffered a higher risk in converting to AD within 3 years, and it added to the growing evidence that the APOE ε4 allele is a reliable genetic risk factor for AD progression.

### Significant Effect of Comprehensive Analysis

Finally, by combining significant neuroimaging factors and clinical variables such as ADAS-cog scores, CDR-SB scores and APOE genotype, the comprehensive Cox model provided a more sufficient investigation of MCI progression, revealing that MCI individuals with reduced gray matter volume in a temporal lobe-related network (IC_06) based on structural MRI, low glucose metabolism in the posterior DMN (IC_27) based on FDG-PET, positive APOE ε4-status, increased ADAS-cog scores and CDR-SB scores were more likely to convert to AD within 36 months after baseline than others, as shown in **Table 2**.

As the ROC results indicated, a combination of neuroimaging factors and clinical variables led to a higher AUC than either neuroimaging factors or clinical variables alone that could be more precise for estimating the risk associated with MCI progression. In other words, the inclusion of multiple types of risk factors would increase the predictive power of the Cox model. While the prediction accuracy was only improved moderately comparing to that using clinical variables alone. Our results seemed to be indicative of relative low sensitivity of applying spatial covarying features, a topic definitely worth further investigation especially with the use of the ICA method.

### Methodological Considerations

Although previous studies have examined multiple biomarkers as predictors for MCI stage, our study provided a perspective by considering brain networks extracted by ICA as predictors and has incorporated neuroimaging factors, genetics, sociodemographic and cognitive variables into the Cox model analysis to assess the progression of MCI. Prior ROI-based analyses have emphasized specific brain regions such as the hippocampus of structural MRI, which exhibited histopathological changes at early stages of AD (Jack et al., 1999; Li et al., 2008; Shi et al., 2009). These analyses relied on a priori knowledge without considering the co-variation of neurological changes in different regions of the human brain. In contrast to the ROI analysis method, which could be viewed as hypothesis driven, ICA is a more objective data-driven approach that does not require the need for any prior information. Moreover, the outcome of the exploratory ICA procedure can be used as a model for independent new data with corresponding hypothesis to be tested. With independence among the ICs, the subsequent inclusion of them in Cox models could avoid the disturbance of multicollinearity, which is sometimes otherwise present among the predictors. Additionally, it is difficult to implement whole brain or voxel-level analyses because of the enormous number of univariate models constructed per voxel, the scattered clusters of significant voxels, and the ill-posed problems of multivariate methods [the number of samples *n* is smaller than the number of variables (voxels) *p*] with lower reliability (Good et al., 2001; Betting et al., 2006; Agosta et al., 2007; Vemuri et al., 2011; Zeifman et al., 2015). However, ICA-based Cox model analyses take the covariance information of voxels into consideration and reduce the number of computations.

### Limitations

A limitation of the present study is the relatively short follow-up period of the MCI participants. MCI individuals were followed for 3 years, as in most previous studies on MCI subjects. Another limitation of the current study is the lack of other imaging data modalities, such as Aβ PET and functional MRI (fMRI). A proportion of MCI samples did not have baseline data of other modalities available in this study. Future studies based on more imaging modalities are needed to assess the risk effects of different combinations of biomarkers on MCI progression to AD. Considering multi-modal data from a larger number of MCI-c subjects who were visited up for a longer period of time might contribute to better performance. In addition, we used ICA in this study for the data of each imaging modality separately to extract the modality specific network. Thus, the ICA itself did not integrate the multi-modal data. Rather, the outcome of the separate ICAs served as joint input to the subsequent Cox procedure. In doing so, the fusion of multi-modality images in feature extraction was not at this stage. As an extension of ICA, Joint ICA can fuse two-modality neuroimaging data and obtain joint ICs (joint sources) sharing the same mixing coefficients (Calhoun et al., 2006). From this perspective, implementing Joint ICA to extract substantially optimized ICs is more likely to further improve the statistical power and deserve a more careful investigation in MCI progression.

## Conclusion

In summary, our results suggested that a combination of ICA and Cox model analyses could be successfully used in survival data analysis to predict MCI progression. Furthermore, our findings indicated that neuroimaging factors, together with clinical variables, can effectively predict the time to progression from MCI to AD. This work offered a network-based perspective in AD-related survival analysis and might be useful in future research.

## Author Contributions

KL, KC, LY, and XG conceived and designed the experiments. KL performed the experiments. KL and XG analyzed the data. KC and LY contributed reagents/materials/analysis tools. KL and XG wrote the paper.

## Conflict of Interest Statement

The authors declare that the research was conducted in the absence of any commercial or financial relationships that could be construed as a potential conflict of interest.

## Footnotes

1 adni.loni.usc.edu

2 http://www.fil.ion.ucl.ac.uk/spm/

3 http://dbm.neuro.uni-jena.de/vbm8

4 http://mialab.mrn.org/software/fit/index.html
