# Supplementary material for: Prediction of Mild Cognitive Impairment Conversion Using a Combination of Independent Component Analysis and the Cox Model
Source: Front Hum Neurosci. 2017 Feb 6;11:33. doi: 10.3389/fnhum.2017.00033 (PMC5292818; doi:10.3389/fnhum.2017.00033)
Supplement: Supplementary file 1 [file Table_1.docx]

Supplementary Material

Prediction of Mild Cognitive Impairment Conversion Using a Combination of Independent Component Analysis and the Cox Model

Ke Liu, Kewei Chen, Li Yao, Xiaojuan Guo*

*** Correspondence:** Xiaojuan Guo: gxj@bnu.edu.cn

# Supplementary Table 1 IDs of 475 ADNI Subjects used in our study

| No. | Subject ID | No. | Subject ID | No. | Subject ID | No. | Subject ID | No. | Subject ID |
| --- | --- | --- | --- | --- | --- | --- | --- | --- | --- |
| 1 | 002_S_1155 | 2 | 002_S_2073 | 3 | 003_S_0908 | 4 | 003_S_1074 | 5 | 003_S_1122 |
| 6 | 005_S_0546 | 7 | 005_S_2390 | 8 | 007_S_0698 | 9 | 007_S_2394 | 10 | 009_S_1030 |
| 11 | 010_S_0422 | 12 | 011_S_2274 | 13 | 012_S_4012 | 14 | 012_S_4128 | 15 | 013_S_1186 |
| 16 | 014_S_2185 | 17 | 018_S_0080 | 18 | 018_S_2155 | 19 | 021_S_0178 | 20 | 021_S_0424 |
| 21 | 021_S_0626 | 22 | 022_S_0961 | 23 | 022_S_2167 | 24 | 022_S_2379 | 25 | 027_S_0408 |
| 26 | 027_S_1045 | 27 | 029_S_1218 | 28 | 031_S_0867 | 29 | 032_S_0718 | 30 | 032_S_2119 |
| 31 | 032_S_2247 | 32 | 035_S_0033 | 33 | 035_S_0292 | 34 | 035_S_2061 | 35 | 036_S_0673 |
| 36 | 036_S_0945 | 37 | 036_S_2378 | 38 | 037_S_0150 | 39 | 037_S_0377 | 40 | 037_S_1421 |
| 41 | 041_S_0679 | 42 | 041_S_1418 | 43 | 041_S_4004 | 44 | 052_S_1346 | 45 | 053_S_0389 |
| 46 | 053_S_0919 | 47 | 053_S_2396 | 48 | 057_S_0464 | 49 | 067_S_2195 | 50 | 067_S_2196 |
| 51 | 067_S_2304 | 52 | 068_S_2168 | 53 | 072_S_2037 | 54 | 072_S_2072 | 55 | 072_S_2083 |
| 56 | 072_S_2093 | 57 | 072_S_2116 | 58 | 072_S_2164 | 59 | 073_S_0746 | 60 | 073_S_2153 |
| 61 | 073_S_2191 | 62 | 073_S_2225 | 63 | 073_S_2264 | 64 | 082_S_2121 | 65 | 082_S_2307 |
| 66 | 094_S_2238 | 67 | 094_S_2367 | 68 | 098_S_0160 | 69 | 099_S_0051 | 70 | 099_S_0291 |
| 71 | 099_S_0551 | 72 | 109_S_2200 | 73 | 114_S_0378 | 74 | 114_S_1106 | 75 | 114_S_1118 |
| 76 | 116_S_0361 | 77 | 123_S_0106 | 78 | 123_S_2055 | 79 | 123_S_2363 | 80 | 126_S_0709 |
| 81 | 126_S_2360 | 82 | 127_S_0112 | 83 | 127_S_0925 | 84 | 127_S_2213 | 85 | 128_S_0135 |
| 86 | 128_S_0138 | 87 | 128_S_0200 | 88 | 128_S_0205 | 89 | 128_S_0225 | 90 | 128_S_0608 |
| 91 | 128_S_2002 | 92 | 128_S_2036 | 93 | 128_S_2045 | 94 | 128_S_2123 | 95 | 128_S_2130 |
| 96 | 128_S_2220 | 97 | 129_S_1246 | 98 | 130_S_0285 | 99 | 130_S_2373 | 100 | 137_S_0481 |
| 101 | 137_S_0722 | 102 | 137_S_0800 | 103 | 137_S_0994 | 104 | 137_S_1414 | 105 | 141_S_2333 |
| 106 | 153_S_2109 | 107 | 153_S_2148 | 108 | 941_S_2060 | 109 | 002_S_1268 | 110 | 002_S_4171 |
| 111 | 003_S_1057 | 112 | 005_S_0222 | 113 | 006_S_0675 | 114 | 006_S_1130 | 115 | 006_S_4515 |
| 116 | 007_S_0101 | 117 | 007_S_0128 | 118 | 007_S_0344 | 119 | 009_S_4324 | 120 | 009_S_4530 |
| 121 | 009_S_4958 | 122 | 010_S_0161 | 123 | 010_S_0904 | 124 | 011_S_0326 | 125 | 011_S_0362 |
| 126 | 011_S_0861 | 127 | 011_S_1282 | 128 | 011_S_4366 | 129 | 012_S_1033 | 130 | 012_S_4094 |
| 131 | 013_S_0240 | 132 | 013_S_0325 | 133 | 013_S_0860 | 134 | 013_S_4595 | 135 | 014_S_4058 |
| 136 | 014_S_4079 | 137 | 014_S_4263 | 138 | 014_S_4668 | 139 | 016_S_4584 | 140 | 018_S_0057 |
| 141 | 018_S_0155 | 142 | 019_S_4293 | 143 | 019_S_4680 | 144 | 021_S_0141 | 145 | 021_S_0231 |
| 146 | 021_S_4402 | 147 | 021_S_4857 | 148 | 022_S_1394 | 149 | 023_S_4035 | 150 | 023_S_4243 |
| 151 | 023_S_4502 | 152 | 023_S_4796 | 153 | 024_S_1393 | 154 | 027_S_0256 | 155 | 027_S_0461 |
| 156 | 027_S_4757 | 157 | 029_S_1318 | 158 | 031_S_0294 | 159 | 031_S_4005 | 160 | 031_S_4042 |
| 161 | 031_S_4203 | 162 | 032_S_0214 | 163 | 032_S_0978 | 164 | 033_S_0511 | 165 | 033_S_0513 |
| 166 | 033_S_0567 | 167 | 033_S_0723 | 168 | 033_S_0906 | 169 | 035_S_0204 | 170 | 035_S_0997 |
| 171 | 035_S_4114 | 172 | 035_S_4414 | 173 | 035_S_4784 | 174 | 036_S_1135 | 175 | 036_S_1240 |
| 176 | 036_S_4430 | 177 | 036_S_4715 | 178 | 037_S_4015 | 179 | 037_S_4706 | 180 | 041_S_0314 |
| 181 | 041_S_0549 | 182 | 041_S_1010 | 183 | 041_S_1412 | 184 | 041_S_1423 | 185 | 052_S_0671 |
| 186 | 053_S_4661 | 187 | 057_S_0941 | 188 | 057_S_1007 | 189 | 057_S_1217 | 190 | 057_S_2398 |
| 191 | 057_S_4888 | 192 | 062_S_1299 | 193 | 067_S_4918 | 194 | 068_S_2248 | 195 | 068_S_2316 |
| 196 | 072_S_4057 | 197 | 072_S_4102 | 198 | 072_S_4131 | 199 | 094_S_1398 | 200 | 094_S_2216 |
| 201 | 094_S_4162 | 202 | 098_S_2047 | 203 | 099_S_0054 | 204 | 100_S_0892 | 205 | 100_S_0930 |
| 206 | 116_S_1243 | 207 | 116_S_4167 | 208 | 123_S_4096 | 209 | 126_S_0708 | 210 | 126_S_0865 |
| 211 | 126_S_1077 | 212 | 126_S_4458 | 213 | 126_S_4507 | 214 | 126_S_4675 | 215 | 126_S_4712 |
| 216 | 127_S_0394 | 217 | 127_S_1427 | 218 | 127_S_4240 | 219 | 127_S_4928 | 220 | 128_S_0947 |
| 221 | 128_S_1043 | 222 | 130_S_0289 | 223 | 130_S_4250 | 224 | 130_S_4415 | 225 | 130_S_4542 |
| 226 | 135_S_4406 | 227 | 135_S_4689 | 228 | 136_S_0695 | 229 | 136_S_4189 | 230 | 137_S_4596 |
| 231 | 137_S_4815 | 232 | 141_S_4426 | 233 | 941_S_1295 | 234 | 941_S_1311 | 235 | 153_S_4172 |
| 236 | 137_S_4211 | 237 | 137_S_4672 | 238 | 135_S_5275 | 239 | 135_S_5015 | 240 | 135_S_4954 |
| 241 | 135_S_4863 | 242 | 135_S_4676 | 243 | 135_S_4657 | 244 | 131_S_5138 | 245 | 130_S_5059 |
| 246 | 130_S_5006 | 247 | 130_S_4997 | 248 | 130_S_4990 | 249 | 130_S_4984 | 250 | 130_S_4982 |
| 251 | 130_S_4971 | 252 | 130_S_4730 | 253 | 130_S_4660 | 254 | 130_S_4641 | 255 | 130_S_4589 |
| 256 | 128_S_5123 | 257 | 128_S_4792 | 258 | 128_S_4774 | 259 | 128_S_4772 | 260 | 127_S_5095 |
| 261 | 127_S_5067 | 262 | 127_S_5058 | 263 | 127_S_5056 | 264 | 127_S_5028 | 265 | 127_S_4992 |
| 266 | 127_S_4940 | 267 | 127_S_4500 | 268 | 126_S_4686 | 269 | 126_S_4494 | 270 | 123_S_4526 |
| 271 | 116_S_4732 | 272 | 116_S_4625 | 273 | 116_S_4338 | 274 | 116_S_4209 | 275 | 116_S_4195 |
| 276 | 100_S_5106 | 277 | 099_S_4994 | 278 | 098_S_4215 | 279 | 098_S_4201 | 280 | 094_S_4737 |
| 281 | 082_S_5184 | 282 | 073_S_4853 | 283 | 070_S_4719 | 284 | 070_S_4692 | 285 | 068_S_4968 |
| 286 | 068_S_4859 | 287 | 067_S_5205 | 288 | 067_S_4728 | 289 | 053_S_5208 | 290 | 053_S_5070 |
| 291 | 052_S_5062 | 292 | 051_S_5005 | 293 | 037_S_4879 | 294 | 037_S_4770 | 295 | 037_S_4001 |
| 296 | 036_S_5210 | 297 | 036_S_5112 | 298 | 036_S_5063 | 299 | 036_S_4894 | 300 | 036_S_4820 |
| 301 | 035_S_4783 | 302 | 033_S_5087 | 303 | 033_S_5017 | 304 | 033_S_5013 | 305 | 032_S_4755 |
| 306 | 031_S_4024 | 307 | 027_S_4964 | 308 | 027_S_4962 | 309 | 027_S_4802 | 310 | 027_S_4801 |
| 311 | 024_S_5054 | 312 | 024_S_4280 | 313 | 024_S_4223 | 314 | 023_S_5241 | 315 | 023_S_5120 |
| 316 | 023_S_4501 | 317 | 021_S_4924 | 318 | 021_S_4718 | 319 | 019_S_5019 | 320 | 019_S_5012 |
| 321 | 019_S_4549 | 322 | 019_S_4477 | 323 | 019_S_4252 | 324 | 016_S_5251 | 325 | 016_S_5057 |
| 326 | 016_S_4887 | 327 | 016_S_4591 | 328 | 016_S_4583 | 329 | 016_S_4353 | 330 | 016_S_4009 |
| 331 | 014_S_4615 | 332 | 013_S_5071 | 333 | 011_S_4912 | 334 | 011_S_4906 | 335 | 011_S_4845 |
| 336 | 011_S_4827 | 337 | 009_S_5037 | 338 | 009_S_5027 | 339 | 007_S_5196 | 340 | 007_S_4911 |
| 341 | 007_S_4568 | 342 | 006_S_4867 | 343 | 006_S_4546 | 344 | 006_S_4192 | 345 | 006_S_4153 |
| 346 | 005_S_5119 | 347 | 005_S_5038 | 348 | 005_S_4910 | 349 | 005_S_4707 | 350 | 003_S_5187 |
| 351 | 003_S_5165 | 352 | 003_S_4373 | 353 | 003_S_4152 | 354 | 003_S_4136 | 355 | 002_S_5018 |
| 356 | 002_S_0295 | 357 | 002_S_0413 | 358 | 002_S_0685 | 359 | 002_S_1261 | 360 | 002_S_1280 |
| 361 | 002_S_4213 | 362 | 002_S_4225 | 363 | 002_S_4262 | 364 | 002_S_4270 | 365 | 003_S_0981 |
| 366 | 003_S_4081 | 367 | 003_S_4119 | 368 | 003_S_4288 | 369 | 003_S_4350 | 370 | 003_S_4555 |
| 371 | 003_S_4644 | 372 | 003_S_4872 | 373 | 003_S_4900 | 374 | 005_S_0553 | 375 | 006_S_0498 |
| 376 | 006_S_4449 | 377 | 006_S_4485 | 378 | 007_S_1206 | 379 | 007_S_4387 | 380 | 007_S_4488 |
| 381 | 007_S_4516 | 382 | 007_S_4620 | 383 | 007_S_4637 | 384 | 009_S_0751 | 385 | 009_S_0842 |
| 386 | 009_S_4388 | 387 | 009_S_4612 | 388 | 010_S_4345 | 389 | 011_S_0021 | 390 | 011_S_0023 |
| 391 | 011_S_4105 | 392 | 011_S_4120 | 393 | 011_S_4222 | 394 | 011_S_4278 | 395 | 012_S_4026 |
| 396 | 012_S_4545 | 397 | 013_S_4579 | 398 | 013_S_4580 | 399 | 013_S_4616 | 400 | 014_S_4080 |
| 401 | 014_S_4093 | 402 | 014_S_4401 | 403 | 014_S_4576 | 404 | 014_S_4577 | 405 | 016_S_4121 |
| 406 | 016_S_4638 | 407 | 016_S_4688 | 408 | 016_S_4951 | 409 | 016_S_4952 | 410 | 018_S_0055 |
| 411 | 018_S_4349 | 412 | 018_S_4399 | 413 | 018_S_4400 | 414 | 019_S_4367 | 415 | 019_S_4835 |
| 416 | 021_S_0984 | 417 | 021_S_4254 | 418 | 021_S_4335 | 419 | 021_S_4421 | 420 | 021_S_4558 |
| 421 | 022_S_0130 | 422 | 022_S_4173 | 423 | 022_S_4196 | 424 | 022_S_4266 | 425 | 022_S_4291 |
| 426 | 022_S_4320 | 427 | 023_S_0926 | 428 | 023_S_1190 | 429 | 023_S_4020 | 430 | 023_S_4164 |
| 431 | 023_S_4448 | 432 | 024_S_4084 | 433 | 024_S_4158 | 434 | 029_S_4279 | 435 | 029_S_4290 |
| 436 | 029_S_4652 | 437 | 031_S_0618 | 438 | 031_S_4021 | 439 | 031_S_4032 | 440 | 032_S_0479 |
| 441 | 032_S_4348 | 442 | 032_S_4386 | 443 | 032_S_4429 | 444 | 032_S_4921 | 445 | 033_S_0920 |
| 446 | 033_S_1016 | 447 | 033_S_4177 | 448 | 033_S_4179 | 449 | 033_S_4508 | 450 | 035_S_4082 |
| 451 | 035_S_4464 | 452 | 037_S_4028 | 453 | 037_S_4410 | 454 | 041_S_4037 | 455 | 041_S_4060 |
| 456 | 041_S_4200 | 457 | 041_S_4427 | 458 | 067_S_0257 | 459 | 070_S_4856 | 460 | 070_S_5040 |
| 461 | 072_S_0315 | 462 | 072_S_4103 | 463 | 073_S_0089 | 464 | 073_S_0311 | 465 | 073_S_4382 |
| 466 | 073_S_4393 | 467 | 073_S_4559 | 468 | 073_S_4739 | 469 | 073_S_4762 | 470 | 082_S_4090 |
| 471 | 082_S_4208 | 472 | 082_S_4224 | 473 | 082_S_4339 | 474 | 082_S_4428 | 475 | 094_S_4234 |

* No.1 – 108: MCI-nc group; No.109 – 234: MCI-c group; No.235 – 355: AD group; No.356 – 475: NC group
